# Postoperative changes in circulating brain injury biomarkers in relation to long-term fatigue and cognitive outcomes after surgery for nonfunctioning pituitary adenomas

**DOI:** 10.1007/s11102-026-01720-7

**Published:** 2026-07-01

**Authors:** Kristin Eyglóardóttir, Thomas Skoglund, Victor Hantelius, Oskar Ragnarsson, David Krabbe, Katharina S Sunnerhagen, Sofie Jakobsson, Henrik Zetterberg, Gudmundur Johannsson, Tobias Hallén

**Affiliations:** 1 Department of Neurosurgery, Sahlgrenska University Hospital, Gothenburg, Sweden; 2 Department of Clinical Neuroscience, Institute of Neuroscience and Physiology, The Sahlgrenska Academy, University of Gothenburg, Gothenburg, Sweden; 3 Department of Internal Medicine and Clinical Nutrition, Institute of Medicine, The Sahlgrenska Academy, University of Gothenburg, Gothenburg, Sweden; 4 Department of Endocrinology, Sahlgrenska University Hospital, Gothenburg, Sweden; 5 Department of Rehabilitation Medicine, Sahlgrenska University Hospital, Gothenburg, Sweden; 6 Institute of Health and Care Sciences, The Sahlgrenska Academy, University of Gothenburg, Gothenburg, Sweden; 7 Wallenberg Centre for Molecular and Translational Medicine, Institute of Medicine, Sahlgrenska Academy, Gothenburg, Sweden; 8 Centre for Person-Centred Care (GPCC), University of Gothenburg, Gothenburg, Sweden; 9 Department of Psychiatry and Neurochemistry, Institute of Neuroscience and Physiology, The Sahlgrenska Academy, University of Gothenburg, Gothenburg, Mölndal, Sweden; 10 Clinical Neurochemistry Laboratory, Sahlgrenska University Hospital, Mölndal, Sweden; 11 Department of Pathology and Laboratory Medicine, University of Wisconsin School of Medicine and Public Health, Madison, WI USA; 12 Wisconsin Alzheimer’s Disease Research Center, School of Medicine and Public Health, University of Wisconsin, University of Wisconsin-Madison, Madison, WI USA; 13 Department of Neurodegenerative Disease, UCL Institute of Neurology, Queen Square, London, UK; 14 UK Dementia Research Institute at UCL, London, UK; 15 Centre for Brain Research, Indian Institute of Science, Bangalore, India

**Keywords:** Brain injury biomarkers, Nonfunctioning pituitary adenomas, PitNET, Pituitary tumors, GFAP, NfL, Tau

## Abstract

**Purpose:**

Circulating brain injury biomarker levels increase after transsphenoidal surgery for pituitary tumors. However, their relationship with long-term fatigue and cognitive outcomes remains unclear. We investigated whether postoperative changes in glial fibrillary acidic protein (GFAP), neurofilament light chain (NfL), and tau are associated with fatigue and cognitive outcomes in patients with nonfunctioning pituitary adenomas (NFPAs).

**Methods:**

This prospective cohort study included 68 patients with NFPAs undergoing endoscopic transsphenoidal surgery. Plasma GFAP, NfL, and tau were measured pre- and postoperatively using ultrasensitive assays. Fatigue was assessed using the Multidimensional Fatigue Inventory and cognitive performance with the Repeatable Battery for the Assessment of Neuropsychological Status preoperatively and at 12-months postsurgery. Clinically meaningful changes were defined as ≥ 0.5 standard deviations from baseline.

**Results:**

All biomarkers increased significantly after surgery, with tau peaking immediately, GFAP on day 1, and NfL on day 5 (all *p* < 0.01). Patients with preoperative hypothalamic compression showed greater postoperative increases in GFAP and NfL. At 12-months, fatigue improved at the group level, and cognitive performance remained stable. However, individual outcomes were heterogeneous: clinically meaningful worsening was observed for fatigue in 8/58 patients (14%) and for cognitive performance in 6/31 patients (19%). We identified no significant associations between increased postoperative biomarker levels and changes in fatigue or cognitive performance.

**Conclusions:**

Endoscopic transsphenoidal surgery for NFPAs is associated with transient increases in circulating brain injury biomarkers, particularly in patients with hypothalamic compression. Our findings revealed that these elevations were not related to long-term fatigue or cognitive decline, suggesting that they may reflect short-term surgical effects without sustained functional consequences.

**Supplementary Information:**

The online version contains supplementary material available at https://doi.org/10.1007/s11102-026-01720-7.

## Introduction

Pituitary adenomas or pituitary neuroendocrine tumors according to the current World Health Organization classification are among the most common intracranial neoplasms, accounting for 15% to 20% of all intracranial tumors [1–3]. Although benign, their location within the sellar region often leads to endocrine and visual dysfunction. Transsphenoidal surgery remains the preferred treatment approach [4–6].

Although many patients experience improved symptoms after surgery, others report persistent fatigue and neurocognitive impairment [7–10]. This is often attributed to postoperative hormonal deficiencies or suboptimal endocrine replacement. Symptoms may also arise from subtle perioperative injury to suprasellar structures, such as the hypothalamus [7, 10]. However, the underlying mechanisms remain incompletely understood.

There are no established clinical methods for detecting or quantifying surgery-related brain injury. Recent advances in ultrasensitive blood assays, particularly single-molecule array (Simoa) technology, enable precise quantification of circulating markers of astroglial and neuronal damage, including glial fibrillary acidic protein (GFAP), neurofilament light chain (NfL), and tau. Elevated concentrations of these markers are linked to injury severity and clinical outcomes in various neurological conditions, including traumatic brain injury [11–13].

Previous investigations of these biomarkers following transsphenoidal pituitary surgery suggest their potential utility to reflect surgical impact on neural structures [14]. Subsequent studies investigating biomarker release after brain tumor surgery refined our understanding of their temporal dynamics. The findings demonstrated associations with postoperative outcomes, including the volume of ischemia observed in postoperative magnetic resonance imaging (MRI) scans and occurrence of new neurological deficits [15, 16].

The present study investigated the relationship between postoperative changes in circulating GFAP, NfL, and tau and long-term fatigue and cognitive outcomes in patients with nonfunctioning pituitary adenomas (NFPA). The aim was to examine whether these biomarkers are associated with the severity and direction of postoperative fatigue and neurocognitive changes, potentially providing insights into long-term postoperative outcomes and their underlying mechanisms

## Materials and methods

### Patients

This study used data from the Gothenburg Pituitary Tumor (GoPT) study, a prospective cohort enrolling individuals scheduled for pituitary surgery at Sahlgrenska University Hospital, Gothenburg, Sweden. This hospital is the sole neurosurgical service provider for the western region of Sweden, serving a population of ~ 1.9 million inhabitants. Ethical approval for the study was granted by the Regional Ethical Review Board in Gothenburg, Sweden (Dnr: 387 − 15), and written informed consent was obtained from all participants.

From October 2017 to January 2021, 104 individuals were admitted for pituitary surgery and enrolled in the GoPT study. Of these, 68 patients diagnosed with NFPA were included in the final analysis to ensure a homogeneous study population. Comprehensive clinical data were obtained, encompassing preoperative comorbidities, pituitary hormonal status, and radiological evaluation. Associations between postoperative biomarker concentrations, intraoperative hypotension, and new pituitary hormone deficiencies in this cohort were reported separately [17].

### Biomarker analysis

Blood samples were collected the day before surgery, immediately after surgery, and on postoperative day 1 and 5. Samples were collected in EDTA Vacutainer tubes, centrifuged for 10 min at 3,800 rpm, and transferred in 1-mL plasma aliquots to cryotubes. The cryotubes were stored at − 70 °C before transport for analysis at the Clinical Neurochemistry Laboratory at Sahlgrenska University Hospital using ultrasensitive Simoa assays according to previously described methods [14].

Sequential sampling allowed within-subject comparisons across time points, with each patient serving as their own control. For each biomarker, the highest measured postoperative concentration was defined as the peak value and used in subsequent analyses.

### Assessment of fatigue and neurocognition

To assess the level of self-reported fatigue at baseline and 12-months after surgery, the Multidimensional Fatigue Inventory (MFI-20) was utilized [18]. The MFI-20 is a validated survey tool designed to evaluate five dimensions of self-reported fatigue, including assessment of general fatigue, physical fatigue, reduced activity, mental fatigue, and reduced motivation. The score ranges from 20 to 100, with higher scores indicating greater fatigue. We used the total MFI-20 score to provide an overall fatigue assessment.

We defined a change in total MFI-20 score of ≥ 0.5 standard deviations (SD) from baseline as the minimal clinically important difference (MCID) for longitudinal analyses and to determine biomarker correlations [19]. Participants were categorized into three groups: improved (MFI-20 score decrease by at least 0.5 SD), worsened (MFI-20 score increase by at least 0.5 SD), and unchanged.

We used the Repeatable Battery for the Assessment of Neuropsychological Status (RBANS) to assess cognitive status [20]. This is a standardized neuropsychological screening tool used to evaluate attention, memory, language, and visuospatial abilities. To reduce practice effects from repeated assessments, two parallel Swedish versions of the RBANS were used, with different versions administered preoperatively and postoperatively. Assessments were performed by a neuropsychologist (DK) the day before surgery and at 12-months after surgery. RBANS provides a score ranging from 40 to 160, with lower scores indicating worse performance. The MCID was defined as a change in total RBANS score of ≥ 0.5 SD [19]. We categorized patients into three groups according to their cognitive status: improved, unchanged, and worsened.

### Surgical techniques

All patients underwent endoscopic transsphenoidal resection of their pituitary tumor. Anesthesia was induced using propofol in all patients and subsequently maintained with a combination of volatile anesthesia (sevoflurane) and either remifentanil with fentanyl or remifentanil alone.

### Radiology and hormonal evaluation

All participants underwent preoperative MRI on a Philips Medical Systems Achieva dStream 3T scanner (Philips Healthcare, Amsterdam, Netherlands) with gadolinium-enhanced T1-weighted sequences. The extent of suprasellar tumor extension was assessed on coronal MRI scans to evaluate the potential risk of neuronal manipulation during the procedure. This assessment included evaluation of tumor proximity to the hypothalamus and was performed by a neurosurgeon (KE). Tumors were classified as either compressing or not compressing the hypothalamus.

Hormone status was evaluated preoperatively, during the first week postoperatively, then every 3 to 6 months during the first year, and every 6 to 12 months thereafter. Adrenal insufficiency and central hypothyroidism were considered especially relevant to postoperative energy levels and neurocognitive function. In patients with no ongoing glucocorticoid treatment, adrenal insufficiency was considered present at a morning cortisol measurement of < 100 nM. Morning cortisol measurements between 100 nM and 349 nM prompted an intravenous corticotrophin stimulation test. Morning cortisol levels ≥ 350 nM were considered normal. Central hypothyroidism was considered present if free thyroxine was low with inappropriately normal or low thyroid-stimulating hormone (TSH). All hormonal assessments were performed by an endocrinologist (VH).

### Statistical analysis

Patient characteristics are presented as the mean ± SD, median with interquartile range (IQR), or counts and percentages, as appropriate. Non-parametric methods were used for group comparisons to account for the asymmetric distributions of biomarker levels measured before and after surgery. We used absolute increases in postoperative biomarker levels (peak postoperative concentration − preoperative concentration) for the analyses. A Wilcoxon signed-rank test was applied to paired (longitudinal) data, and group comparisons were performed using the Mann–Whitney U test or Kruskal–Wallis test.

For analyses of associations with continuous fatigue and cognitive outcomes, biomarker changes were expressed as log_10_ values (peak postoperative concentration / preoperative concentration) and evaluated using linear regression models with confidence intervals (CIs). Non-parametric correlations were assessed using Spearman’s rank correlation. Sensitivity analyses were performed by repeating key analyses after exclusion of outliers.

All tests were two-tailed, and a *p* < 0.05 was considered statistically significant. Data were analyzed using SPSS (v.29; IBM Corp., Armonk, NY, USA) and visualized using GraphPad Prism (v.10; GraphPad Software, San Diego, CA, USA).

## Results

### Patient demographics and baseline characteristics

Our cohort included 68 patients with NFPA (47 men and 21 women) and a mean age of 63 ± 13 years. Detailed demographic information and clinical characteristics are presented in Table 1.

**Table 1:** Patient characteristics

Patients included in study, No	68
Mean age, years (SD)	63 (13)
Gender, No. (%) Male Female	47 (69) 21 (31)
Preoperative comorbidities, No. (%) Ischemic heart disease Ischemic stroke Diabetes mellitus type II Hypertension Psychiatric illness Heart failure Cancer	7 (10) 4 (6) 9 (13) 26 (38) 6 (9) 0 (0) 4 (6)
Preoperative hormone deficiency, No. (%) Central hypothyroidism (TSH) Secondary adrenal insufficiency (ACTH) Growth hormone deficiency (GH) Diabetes insipidus Hypogonadotropic hypogonadism	24 (35) 20 (29) 19 (28) 4 (6) 36 (53)
Hypothalamus compression, No. (%) Yes No	19 (28) 49 (72)
Type of anesthesia, No. (%) Sevoflurane/Remifentanil Propofol/Remifentanil Combined	65 (96) 2 (3) 1 (1)
Baseline MFI total score (fatigue), median (IQR)	56 (38–75)
Baseline RBANS score (cognition), median (IQR)	87 (75–104)

### Biomarker dynamics related to surgery

**Fig. 1:**
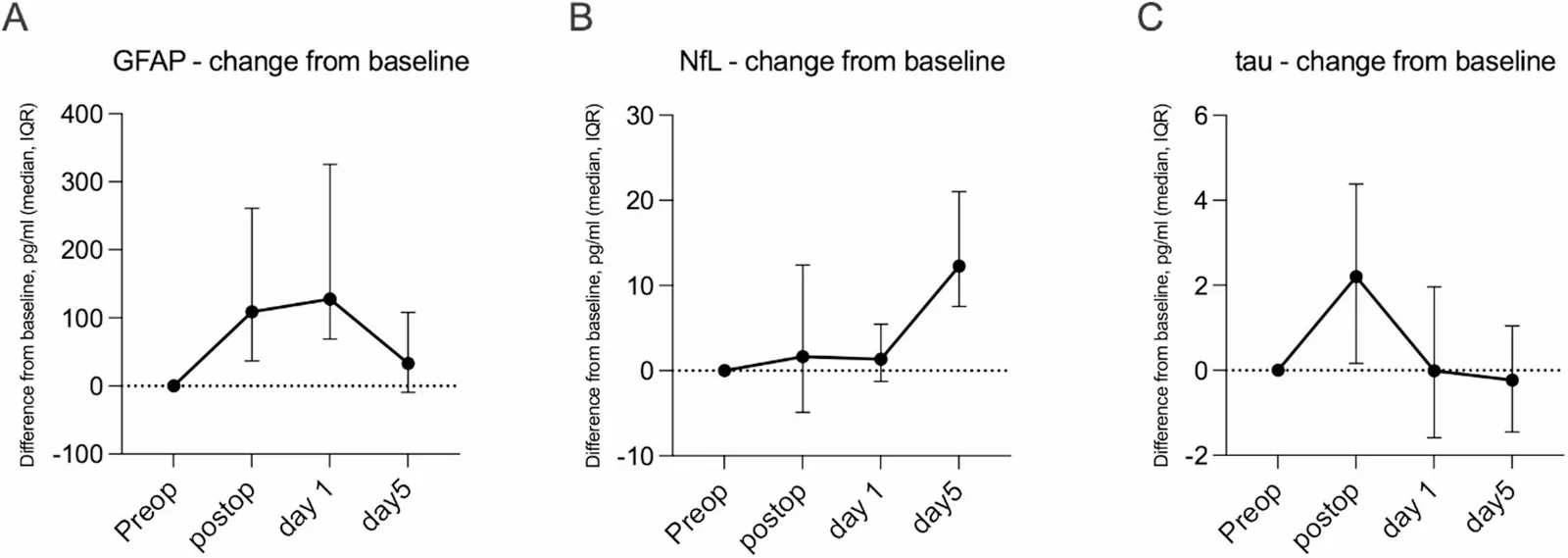
Postoperative changes in circulating brain injury biomarkers following transsphenoidal surgery. Median (IQR) changes from baseline in circulating (A) GFAP, (B) NfL, and (C) tau. GFAP peaked on postoperative day 1, NfL on postoperative day 5, and tau immediately after surgery

Table 2; Fig. 1 show baseline (preoperative) levels and postoperative changes in GFAP, NfL and tau. GFAP concentration peaked on postoperative day 1 (*p* < 0.001), NfL concentration peaked on day 5 (*p* < 0.001), and tau concentration peaked immediately after surgery (*p* = 0.001). All subsequent postoperative biomarker analyses were performed using the respective peak increases relative to baseline concentrations.

There were no significant differences in baseline biomarker levels between men and women. Baseline levels showed positive correlations with age for GFAP (*r* = 0.53; *p* < 0.001) and NfL (*r* = 0.59; *p* < 0.001) but not for tau. No significant correlations were observed between age and postoperative peak increases for any of the biomarkers.

**Table 2:** Longitudinal changes in plasma concentration of glial fibrillary acidic protein (GFAP), neurofilament light (NfL), and tau after endoscopic transsphenoidal surgery for NFPA

Timepoint	Variable	GFAP		NFL		tau	
		Median (IQR) [no.*,%]	*p*-value	Median (IQR) [no.*,%]	*p*-value	Median (IQR) [no.*,%]	*p*-value
Baseline	Absolut Concentration (pg/ml)	121 (96.3–182) [*n* = 64]	NA	15.7 (11.2–21.4) [*n* = 66]	NA	4.1 (2.8–5.5) [*n* = 66]	NA
Immediately after surgery	Difference from baseline (pg/ml)	109 (36.7–261) [*n* = 27, 42%]	0.001	1,65 (-4.9-12.4) [*n* = 28, 42%]	0.198	2,2 (0.2–4.4) [*n* = 28, 42%]	0.001
Day 1	Difference from baseline (pg/ml)	128 (69–326) [*n* = 62, 97%]	0.001	1.3 (-1.25-5.5) [*n* = 65, 99%]	0.005	-0.01 (-1.6-2) [*n* = 65, 99%]	0.636
Day 5	Difference from baseline (pg/ml)	33 (-9.5-108) [*n* = 58, 91%]	0.001	12.3 (7.6–21) [*n* = 60, 91%]	0.001	-0.23 (-1.5-1.1) [*n* = 60, 91%]	0.387

*Numbers and percentages represent the number of available samples at each sampling time point

*GFAP* glial fibrillary acidic protein, *NfL* neurofilament light chain, *NFPA* nonfunctioning pituitary adenoma

### Hypothalamic compression and postoperative biomarker levels

**Fig. 2:**
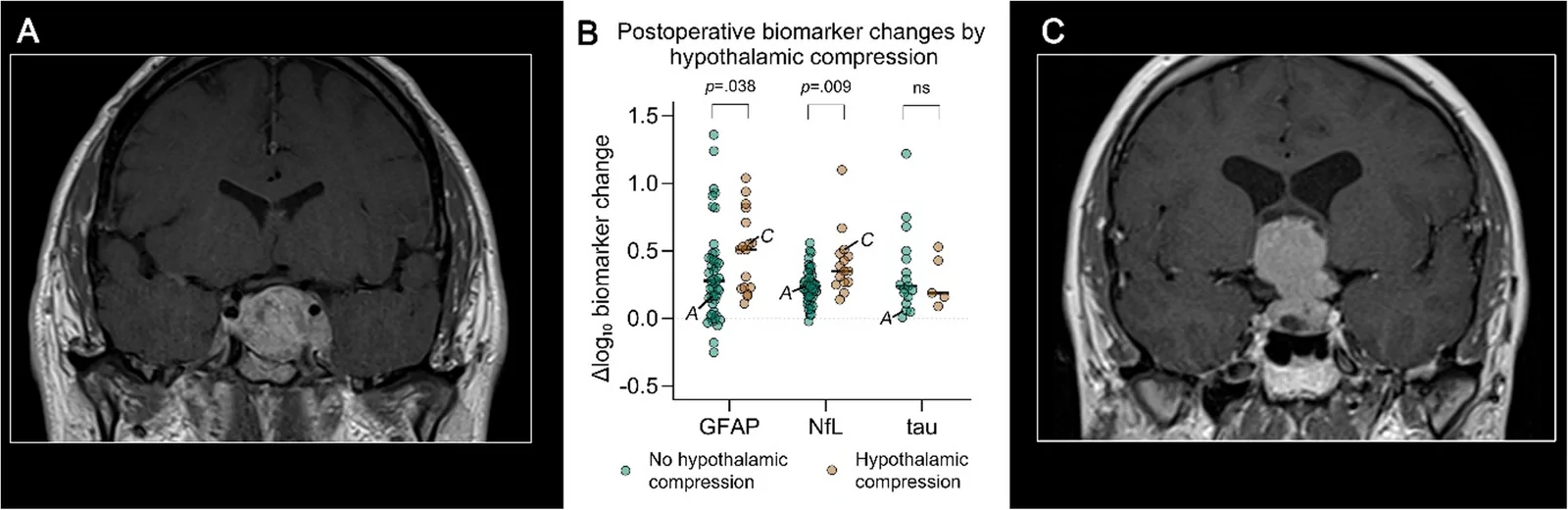
Postoperative biomarker changes related to hypothalamic compression. (A) Preoperative coronal contrast-enhanced T1-weighted MRI of a patient with a large non-functioning pituitary adenoma without hypothalamic compression. (B) Changes in plasma biomarker levels from preoperative to postoperative sampling in patients with and without hypothalamic compression. Each dot represents an individual patient. Black bars indicate medians. Statistical comparisons (Mann–Whitney U test) were performed on absolute biomarker changes, and data are plotted on a log_10_ scale for visualization. Data are from patients represented in the MRI scans in panels (A) and (C). The peak tau blood sample was unavailable for the patient shown in panel (C). (C) Preoperative coronal contrast-enhanced T1-weighted MRI of a patient with pronounced hypothalamic compression

There were no differences in baseline biomarker levels between patients with and without tumors compressing the hypothalamus. Increases in postoperative biomarker levels were significantly higher in the compression group for GFAP on postoperative day 1 (*p* = 0.038) and NfL on postoperative day 5 (*p* = 0.009). No significant difference was observed for tau at its peak concentration immediately after surgery (Fig. 2).

### Patient-reported fatigue

A total of 58 patients completed the MFI-20 assessment both preoperatively and at 12-months postoperatively. The median preoperative total MFI-20 score was 56 (IQR: 38–75), which decreased to 49 (IQR: 30–68) at 12-months (*p* = 0.014). This indicated significantly lower fatigue at the group level at 12 months after surgery.

**Fig. 3:**
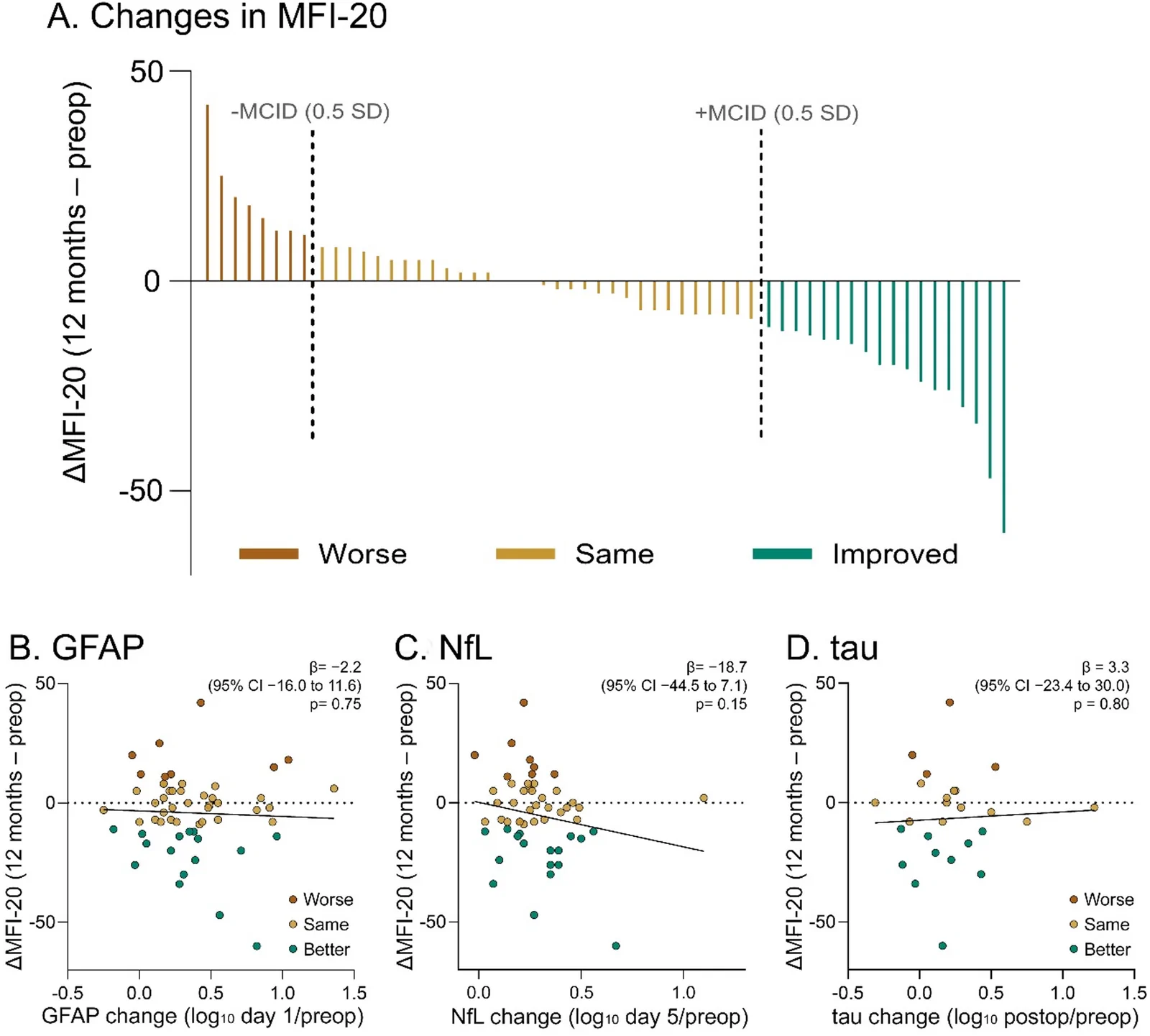
Changes in fatigue and relation to postoperative biomarker levels. (A) Waterfall plot showing individual changes in total MFI-20 score from preoperative assessment to 12 months postoperatively (*n* = 58). The dotted lines indicate the threshold for minimal clinically important difference (MCID; (± 0.5 SD). Negative values indicate lower fatigue scores (improvement). (B–D) Linear regression analysis of postoperative changes in biomarker levels (GFAP, NfL, and tau) and 12-month postoperative changes in MFI-20 score. Changes in biomarker levels are expressed as log_10_ values (peak postoperative concentration/preoperative concentration). Peak postoperative levels corresponded to postoperative day 1 (GFAP), day 5 (NfL), and immediately after surgery (tau). Points are color-coded according to MCID-based outcome categories (worsened, unchanged, improved). Solid lines represent linear regression fits. Regression coefficients (β), 95% confidence intervals, and p-values are shown in each panel

Individual patient measurements revealed that 18 patients (31%) showed improvement, 32 (55%) remained unchanged, and eight (14%) reported increased fatigue relative to baseline based on the MCID criterion. Individual changes in reported fatigue are shown in Fig. 3a.

### Cognitive status

Thirty-one patients completed the RBANS assessment both preoperatively and at 12-months after surgery. The median total RBANS score was 87 (IQR: 74–103) before and 83 (IQR: 72–97) after surgery (*p* = 0.741), indicating no statistically significant change at the group level.

**Fig. 4:**
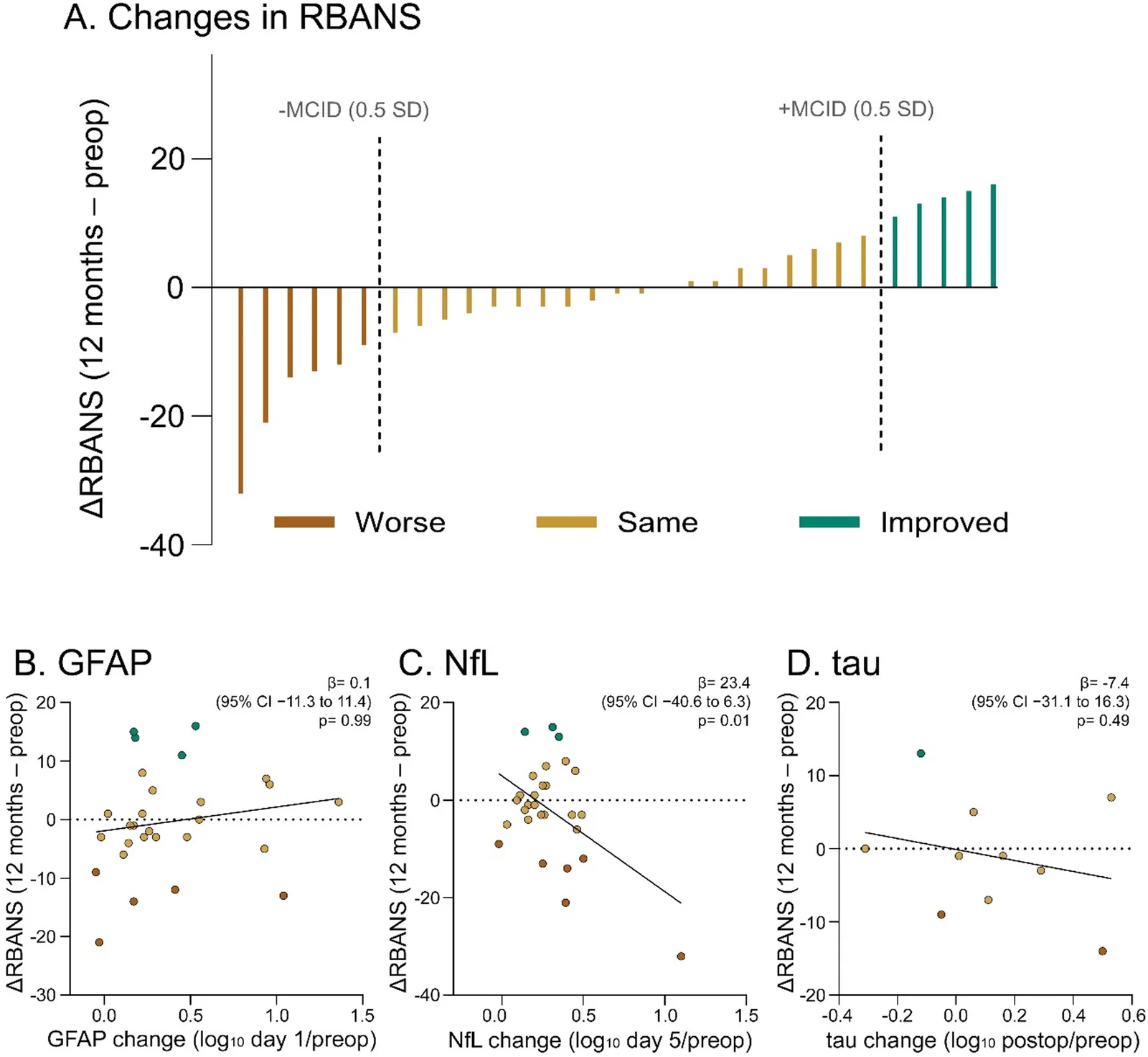
Changes in cognitive outcomes relative to postoperative biomarker levels. (A) Waterfall plot showing individual changes in RBANS score from preoperative assessment to 12-months postoperatively (*n* = 31). The dotted line indicates the threshold for minimal clinically important difference (MCID; (± 0.5 SD). Positive values indicate improvement. (B-D) Linear regression analysis of changes in postoperative biomarker levels (GFAP, NfL, and tau) and 12-month postoperative changes in RBANS score. Peak postoperative levels corresponded to postoperative day 1 (GFAP), day 5 (NfL), and immediately after surgery (tau). Points are color-coded according to MCID-based outcome categories (worsened, unchanged, improved). Solid lines represent linear regression fits

Individual patient measurements identified five patients (16%) that showed improved cognitive function, 20 (65%) that remained unchanged, and six (19%) that showed decreased function based on the MCID criterion. Individual changes in cognitive function are shown in Fig. 4a.

### Relationships between biomarker levels and functional outcomes

Linear regression analyses showed no statistically significant associations between changes in postoperative biomarker levels and changes in MFI-20 outcomes at 12-months after surgery (Fig. 3b–d). Analyses based on predefined MFI-20 categories similarly showed no significant differences in peak postoperative biomarker increases between improved, unchanged, and worsened groups (Online Resource 1, Supplementary Fig. 1).

Linear regression analysis identified a statistically significant negative association between changes in perioperative NfL levels and changes in the RBANS score at 12-months after surgery (β = −23.4, 95% CI: −40.6 to − 6.3; *p* = 0.009) (Fig. 4c). However, this association was not confirmed in non-parametric analysis using Spearman’s rank correlation and was sensitive to the exclusion of a single outlier in the data (Online Resource 1, Supplementary Fig. 2). No significant associations were observed between changes in RBANS scores and changes in perioperative GFAP or tau levels (Fig. 4b–d) Analyses based on predefined RBANS categories similarly showed no significant differences in peak postoperative increases in GFAP, NfL, or tau levels between improved, unchanged, and worsened groups (Online Resource 1, Supplementary Fig. 1).

Five of 48 (10%) patients without preoperative adrenocorticotropic hormone (ACTH) insufficiency developed new ACTH insufficiency, and 15 of 42 (36%) patients without preoperative TSH deficiency developed new TSH insufficiency. Four patients developed both. Patients with any new hormonal deficit (ACTH and/or TSH) did not differ from those without such deficits in terms of changes in fatigue (MFI-20 score) or cognitive performance (RBANS score) (data not shown).

Biomarker change was expressed as log_10_ values (peak postoperative concentration/preoperative concentration). Peak postoperative levels corresponded to day 1 (GFAP), day 5 (NfL), and immediately after surgery (tau). Points are color-coded according to MCID-based outcome categories (worse, unchanged, improved). Solid lines represent linear regression fits. Regression coefficients (β), 95% confidence intervals, and p-values are shown in each panel.

## Discussion

In this prospective study of patients with NFPAs, we observed clear postoperative increases in circulating biomarkers of astroglial and neuronal injury that peaked at different time points after surgery. Patients with preoperative hypothalamic compression by the adenoma displayed significantly greater postoperative increases in GFAP and NfL, whereas no significant difference was observed for tau. We identified no robust associations between increased biomarker levels and changes in patient-reported fatigue or cognitive performance at 12-months after surgery.

These findings suggest that brain injury may occur during transsphenoidal surgery, with a biomarker-release pattern similar to that reported in other neurosurgical procedures [15]. The timing of the peaks in biomarker concentrations was consistent with expected postoperative changes, with tau increasing immediately after surgery, GFAP on postoperative day 1, and NfL on day 5. This temporal pattern is in line with our previous observations in patients undergoing pituitary surgery [14]. The finding that patients with preoperative hypothalamic compression by the adenoma showed higher postoperative increases in GFAP and NfL indicates greater surgical stress in these cases. The postoperative elevations in biomarker levels might reflect short-term effects on suprasellar structures during the procedure.

Previous studies report different findings regarding postoperative fatigue and cognitive outcomes following pituitary adenoma surgery. A systematic review by Alsumali et al. reported limited and methodologically inconsistent evidence concerning cognitive impairment after transsphenoidal surgery [21]. Recent studies have generally shown stable cognitive and fatigue outcomes after transsphenoidal surgery. We previously showed that fatigue levels remained stable at 6-months after surgery [14], and that cognitive performance remained relatively unchanged from pre- to 12-months after surgery [22]. Additionally, a cross-sectional analysis reported lower cognitive scores relative to age-adjusted normative data rather than a postoperative decline [23]. Those studies included patients with both NFPAs and functioning pituitary adenomas. Moreover, two of those cohorts partially overlapped with that used in the present study [22, 23], although the current analysis focused exclusively on patients with NFPAs.

Although group-level results frequently demonstrate small changes in postoperative outcomes, individual postoperative trajectories may be more heterogeneous. Shen et al. [24] identified distinct subgroups with different recovery patterns, including a subset of patients who experienced a relevant deterioration in fatigue over time. In the present study, fatigue and cognitive outcomes were largely consistent with previous reports. At the group-level, fatigue measured using the MFI-20 improved modestly at 12 months after surgery, and cognitive performance assessed with the RBANS remained unchanged from preoperative levels. However, examination of individual trajectories showed more variation. Almost 20% of patients showed decreased cognitive performance, and a smaller subset demonstrated increased fatigue. These individual declines were not reflected in group-level averages but may represent a clinically relevant subgroup that warrants further investigation.

Despite clear postoperative increases in GFAP, NfL, and tau, we found no robust associations between biomarker changes and postoperative outcomes. Although linear regression identified an initial association between change in NfL level and 12-month cognitive outcome, this was not confirmed in non-parametric analysis. This result was also sensitive to the exclusion of a single patient with an exceptionally complex surgical course, discussed further below. These findings suggest that the observed elevations in biomarker levels in most patients reflected transient surgical effects rather than lasting brain injury with functional consequences.

The patient whose NfL level represented an outlier in the data had a markedly prolonged operative time (~ 2-fold longer than the cohort median) and showed worse cognitive outcomes at 12-months after surgery. Although results from a single patient must be interpreted with caution, this observation is consistent with our previous findings demonstrating that higher NfL levels following extensive brain tumor surgery are associated with greater neurological impact and worse postoperative outcomes [15, 16]. This observation suggests that increased NfL levels may reflect greater neuronal stress in cases of unusually complex surgery.

A potential confounder in the interpretation of postoperative fatigue and cognitive outcomes is the development of new hormonal deficiencies. Previous studies show that hypopituitarism and multiple pituitary hormone deficiencies are associated with impaired quality of life and increased fatigue in patients treated for NFPAs [9]. In the present study, 20 patients developed new postoperative ACTH or TSH insufficiency, both conditions that could potentially influence energy levels and cognitive function. However, comparison of changes in the MFI-20 and RBANS scores between patients with and without new hormonal deficits 12 months after surgery revealed no differences. Notably, postoperative hormonal deficiencies were identified early and promptly treated with adequate hormone replacement, which likely minimized their long-term impact.

A possible explanation for the elevated biomarker levels is direct surgical injury to the pituitary gland. Both GFAP and tau are present in the posterior pituitary gland [25, 26], and GFAP is also present in the anterior pituitary gland [27]. The presence of NfL in pituitary tissue is less well established, although its presence in the posterior lobe is biologically plausible due to the dense population of hypothalamic axons terminating there. Thus, the increased biomarker levels observed in association with suprasellar extension may reflect a greater degree of pituitary gland injury during surgery for larger tumors. Notably, we could not distinguish whether the circulating biomarkers measured in this study were released from pituitary tissue or from adjacent brain structures. Therefore, the observed biomarker elevations may reflect injury to both tissues. In a separate analysis of this cohort, postoperative biomarker elevations were associated with new pituitary hormone deficiencies, consistent with direct pituitary injury as a potential source of biomarker release [17]. Additionally, GFAP expression has been reported in folliculo-stellate cells in a subset of pituitary adenomas [28–29]. Consequently, we cannot completely exclude a minor contribution of tumor-derived GFAP to the observed postoperative elevations. In contrast, to the best of our knowledge, no studies have reported NfL or tau expression in pituitary adenoma tissue.

It has been suggested that increases in brain injury biomarkers may be related to anesthesia. However, studies in healthy volunteers undergoing general anesthesia without surgery showed no elevations in GFAP, tau, or NFL. Similar findings were reported after functional endoscopic sinus surgery, a procedure comparable to endoscopic transsphenoidal surgery without proximity to neural structures [30–32]. Together, these data indicate that anesthesia and sinonasal surgery without central nervous system involvement have minimal impact on the release of brain injury biomarkers.

Strength and Limitations.

A strength of this study is its prospective design, which allowed for systematic and standardized data collection. The primary limitation is the small sample size. In addition, the number of patients undergoing RBANS assessment was lower than that for MFI-20, as neuropsychological testing was introduced.

at a later stage. Additionally, evaluation of fatigue and cognitive performance is inherently challenging based on their dependence on the fluctuating and multifactorial states of each patient, as well as the influence of external factors and hormonal variations. The development of new hormone deficiencies could have represented a potential confounding factor; however, the sample size in our study may be too small to provide sufficient statistical power to detect such effects. Restricting our patient group to only NFPA has, to a certain extent, decreased the possible confounding effect of hormonal disturbances, as seen in acromegaly and Cushing’s disease. Regarding the timing of our measurements, NfL reportedly demonstrates slow biomarker dynamics, suggesting that we may have missed the peak NfL concentration [16]. Our application of a 0.5 SD threshold is in line with its common use to define MCID when condition-specific values are unavailable. Although this approximates a minimal meaningful change across many health outcomes [19], it has not been validated for patients with NFPAs.

## Conclusions

In this prospective cohort of patients undergoing endoscopic surgery for NFPAs, we observed significant postoperative increases in the circulating brain injury biomarkers GFAP, NfL and tau. Postoperative increases in GFAP and NfL were associated with preoperative radiological evidence of hypothalamic tumor compression, suggesting a higher degree of perioperative astroglial and neuronal stress in tumors with suprasellar extension. However, we did not observe consistent associations between postoperative biomarker changes and long-term fatigue or cognitive outcomes 12 months after surgery. Taken together, our findings suggest that the postoperative biomarker elevations primarily reflect transient perioperative tissue stress rather than mechanisms underlying long-term fatigue or cognitive decline. Accordingly, we found no evidence supporting their use as prognostic biomarkers for long-term fatigue and cognitive outcomes in this patient population.

## Supplementary Information

Below is the link to the electronic supplementary material.

Supplementary Material 1 (DOCX 453 KB)

## Data Availability

No datasets were generated or analysed during the current study.

## Declarations

GJ has served as a consultant for Crinetics, Novo Nordisk, and Astra Zeneca and has received lecture fees from Novo Nordisk, Pfizer, and Pharmanovia.HZ has served at scientific advisory boards and/or as a consultant for Abbvie, Acumen, Alamar, Alector, Alzinova, ALZpath, Amylyx, Annexon, Apellis, Artery Therapeutics, AZTherapies, Cognito Therapeutics, CogRx, Denali, Eisai, Enigma, LabCorp, Merck Sharp & Dohme, Merry Life, Nervgen, Novo Nordisk, Optoceutics, Passage Bio, Pinteon Therapeutics, Prothena, Quanterix, Red Abbey Labs, reMYND, Roche, Samumed, ScandiBio Therapeutics AB, Siemens Healthineers, Triplet Therapeutics, and Wave; has given lectures sponsored by Alzecure, BioArctic, Biogen, Cellectricon, Fujirebio, LabCorp, Lilly, Novo Nordisk, Oy Medix Biochemica AB, Roche, and WebMD; and is a co-founder of Brain Biomarker Solutions in Gothenburg AB (BBS), which is a part of the GU Ventures Incubator Program, and is a shareholder of CERimmune Therapeutics (outside submitted work).
