# Supplementary material for: Postoperative changes in circulating brain injury biomarkers in relation to long-term fatigue and cognitive outcomes after surgery for nonfunctioning pituitary adenomas
Source: Pituitary. 2026 Jul 1;29(4):111. doi: 10.1007/s11102-026-01720-7 (PMC13323199; doi:10.1007/s11102-026-01720-7)
Supplement: Supplementary file 1 — Supplementary Material 1 (DOCX 453 KB) [file 11102_2026_1720_MOESM1_ESM.docx]

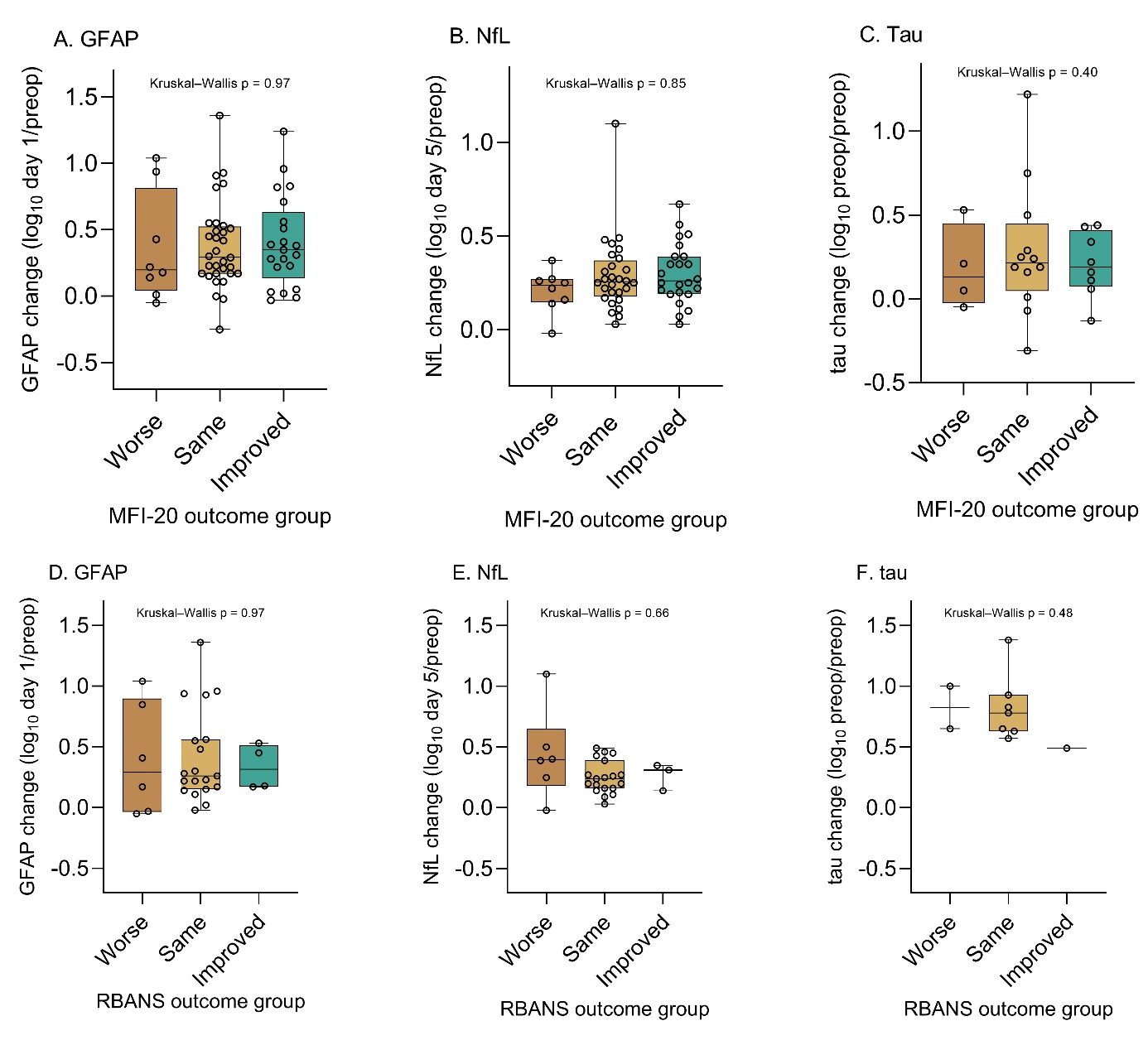


Supplementary Figure 1. Postoperative biomarker changes in relation to categorical fatigue and cognitive outcomes.

(A–C) Peak postoperative changes in GFAP (day 1), NfL (day 5), and tau (immediately postoperative) according to MFI-20 outcome categories (worsened, unchanged, improved).

(D–F) Corresponding analyses according to RBANS outcome categories.

Biomarker change is expressed as log_10_(peak postoperative/preoperative concentration). Boxplots show median and interquartile range; individual data points are overlaid. Group comparisons were performed using the Kruskal–Wallis test on non-log-transformed absolute peak increases. P-values are shown in each panel.


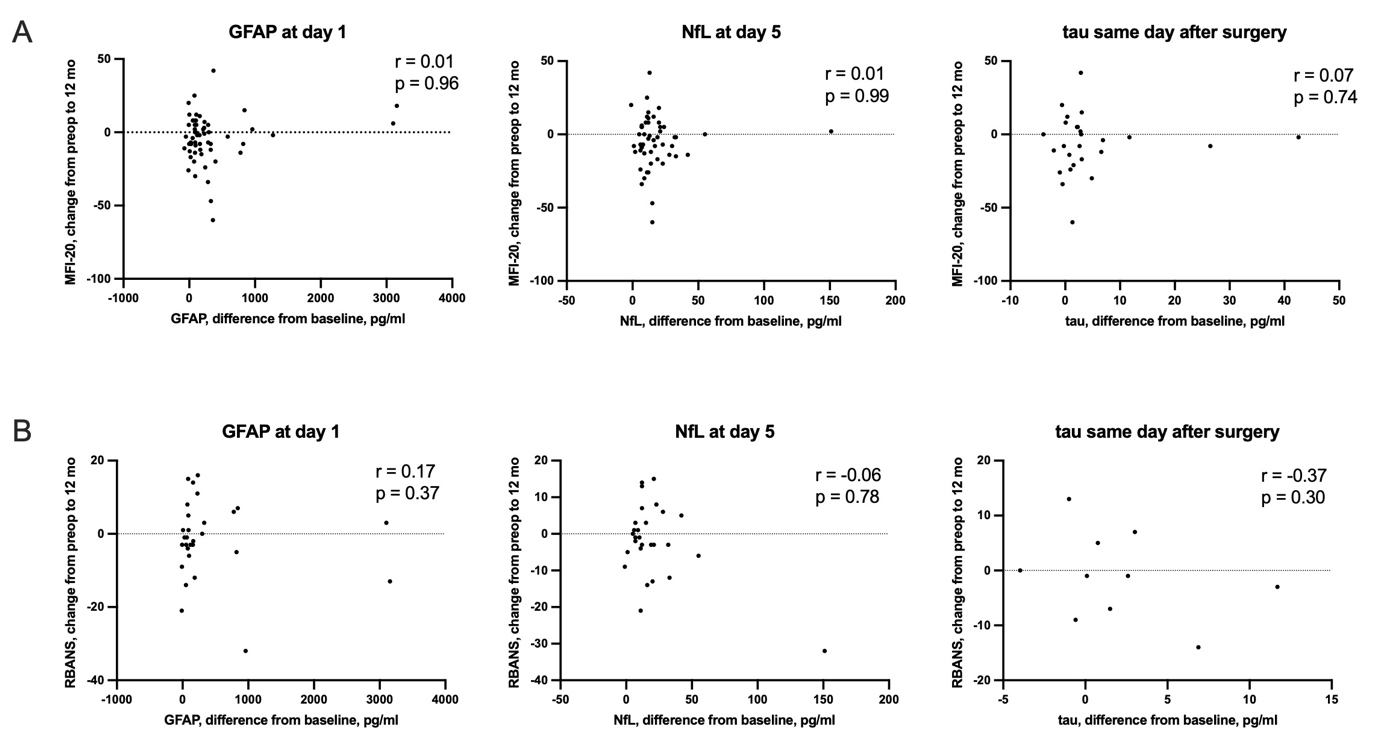


Supplementary Figure 2. Correlations between change in Mental Fatigue Inventory (MFI-20) (A) and RBANS (B) scores and postoperative peak increase in GFAP on day 1, NfL on day 5 and tau immediately after surgery, respectively. Correlations calculated using Spearman’s correlation coefficient.
